# Adverse Childhood Experiences and Sleep Disturbances Among Puerto Rican Young Adults

**DOI:** 10.1001/jamanetworkopen.2024.7532

**Published:** 2024-04-22

**Authors:** Eudora L. Olsen, Ayana K. April-Sanders, Hector R. Bird, Glorisa J. Canino, Cristiane S. Duarte, Shakira F. Suglia

**Affiliations:** 1 Department of Epidemiology, Emory University Rollins School of Public Health, Emory University School of Medicine MD Program, Atlanta, Georgia; 2 Department of Biostatistics and Epidemiology, Rutgers School of Public Health, Piscataway, New Jersey; 3 Department of Psychiatry, Ponce Medical School, Ponce, Puerto Rico; 4 Division of Child and Adolescent Psychiatry, Columbia University Medical Center, New York, New York; 5 Behavioral Sciences Research Institute, University of Puerto Rico Medical School, San Juan, Puerto Rico; 6 New York State Psychiatric Institute, New York; 7 Department of Epidemiology, Emory University Rollins School of Public Health, Atlanta, Georgia

## Abstract

**Importance:**

Sleep quality is a known marker of overall health. Studies suggest that adverse childhood experiences (ACEs) are associated with sleep disturbances among children and adults.

**Objective:**

To examine the association of retrospective and prospective ACEs with sleep quality among a cohort of Puerto Rican young adults from 2 sociocultural contexts.

**Design, Setting, and Participants:**

This prospective cohort study used data from the Boricua Youth Study (BYS), a population-based study representing Puerto Rican children from the South Bronx, New York, and Puerto Rico conducted from August 2000 to August 2003. Participants who were 5 to 9 years of age at enrollment in the BYS and who participated in wave 4 of the BYS took part in the Health Assessment (HA) when they were 18 to 29 years of age, from April 2013 to August 2017. Of the eligible 982 participants, 813 (82.8%) participated in the HA. Statistical analysis was conducted from January 2023 to January 2024.

**Exposures:**

Prospective ACEs measured from parent and youth responses and retrospective ACEs measured among young adults using questions from the validated ACE questionnaire from the original ACEs study conducted by Kaiser Permanente and the Centers for Disease Control and Prevention and published in 1998. Analysis included 8 overlapping items from both questionnaires.

**Outcomes:**

Sleep quality was assessed in the HA with the Pittsburgh Sleep Quality Index. The summary score included 7 components of the Pittsburgh Sleep Quality Index. The hypothesis was formulated after data collection. Sleep quality information was gathered at the same time as retrospective ACEs in the HA.

**Results:**

Of the 813 participants, 438 (53.9%) lived in Puerto Rico as children, 411 (50.6%) identified as female, and the mean (SE) age of participants was 22.9 (0.07) years. After adjusting for sociodemographic factors, retrospective ACEs had a significant association with worse sleep outcomes (β [SE] = 0.29 [0.07]; 95% CI, 0.15-0.44; *P* < .001). Prospective ACEs did not have a significant association with sleep quality, after adjusting for sociodemographic factors (β [SE] = 0.05 [0.10]; 95% CI, –0.14 to 0.24; *P* = .59).

**Conclusions and Relevance:**

This study suggests that there is a significant association between retrospective ACEs and sleep quality among Puerto Rican young adults, after adjusting for sociodemographic factors. Prospective ACEs were not significantly associated with sleep disturbances, after adjusting for sociodemographic factors. Addressing ACEs reported in young adulthood may help reduce sleep disorders.

## Key Points

**Question:**

Are prospectively and retrospectively reported adverse childhood experiences (ACEs) associated with sleep disturbances among Puerto Rican young adults?

**Findings:**

In this cohort study that included 813 Puerto Rican young adults, higher numbers of retrospectively reported ACEs were significantly associated with greater sleep disturbance, while prospectively reported ACEs were not significantly associated with greater sleep disturbance.

**Meaning:**

This study suggests that retrospectively reported ACEs are significantly associated with sleep disturbances among Puerto Rican young adults, after adjusting for sociodemographic factors.

## Teaser

This cohort study examines the association of retrospective and prospective adverse childhood experiences (ACEs) with sleep quality among a cohort of Puerto Rican young adults from 2 sociocultural contexts.

## Introduction

Sleep quality is a known marker of overall health and is a chronic health issue in the US.^1,2^ In addition to the racial and ethnic disparities affecting sleep quality, studies suggest that adverse childhood experiences (ACEs) are associated with sleep disturbances and other chronic diseases among adults.^3,4^ ACEs are defined as stressful or traumatic life events that occur during the first 18 years of life.^5^ It is estimated that approximately 58% of youths in the US have experienced at least 1 or more ACE, with a higher prevalence of ACEs among Black and non-White Hispanic children.^5,6,7^ Our research group previously found a significant association between childhood adversity and poor sleep outcomes among Puerto Rican children.^8^ Although ACEs can be assessed both prospectively during childhood and retrospectively in adulthood, no previous study on ACEs and sleep quality has used both forms of reporting, to our knowledge. Despite the utility of retrospective ACEs and patient preference to be screened, both patients and physicians reported that fewer than 10% of adult patients were asked about ACEs.^9,10^ Furthermore, research has shown low agreement between prospective and retrospective ACEs, suggesting that these 2 forms of reporting may identify distinct groups of people with unique risk factors for various health outcomes.^11,12^

Compared with previous research on ACEs and sleep, our study introduces a new age group to consider: young adults. Young adults, defined as approximately 18 to 26 years of age, are an underrepresented population in research.^13^ Young adulthood is a critical period of complex cognitive and emotional maturation, including increased rates of risky behavior and accidental death.^13^ Despite the high incidence of mental and physical health issues among young adults, this population is less likely to seek health care or undergo routine screening compared with other age groups.^14^

Considering the disparities in research that exist for Puerto Rican young adults, as well as the promising importance of exploring both retrospective and prospective ACEs, our study examines the association of prospective and retrospective ACEs with sleep quality in a geographically diverse population of Puerto Rican young adults. Understanding this association has the potential to inform interventions to address ACEs, improve sleep quality, and reduce health disparities for Puerto Rican young adults.

## Methods

### Study Population

Study participants are from the Boricua Youth Study (BYS), a longitudinal cohort of Puerto Rican children living in the South Bronx, New York, and Puerto Rico from August 2000 to August 2003. Details on the study sampling, design, and procedures can be found elsewhere.^15^ In brief, 2491 Puerto Rican children and adolescents aged 5 to 13 years old were recruited at baseline from South Bronx, New York (n = 1138), and the standard metropolitan area of San Juan and Caguas, Puerto Rico (n = 1353). Eligibility criteria for the BYS included having at least 1 child in the household who was 5 to 13 years of age and at least 1 parent or primary caretaker of Puerto Rican descent. Participant ethnicity was assessed through a questionnaire. The BYS Health Assessment (HA) recruited a subsample of those who were 5 to 9 years of age at enrollment and participated in wave 4 from April 2013 to August 2017. Of the eligible 982 participants, 813 (82.8%) participated in the HA. Data for this study come from those who participated in the HA. The BYS was approved by the institutional review boards at the New York State Psychiatric Institute and the University of Puerto Rico Medical Sciences Campus. Written informed consent was obtained from participants, and interviews were conducted in English and Spanish. This cohort study adheres to the Strengthening the Reporting of Observational Studies in Epidemiology (STROBE) reporting guideline.^16^

### Main Exposure: ACEs

Prospective ACEs are defined as those reported in childhood, and retrospective ACEs are defined as those reported in young adulthood. Prospective ACEs were reported by parents and youths using the 10 items outlined in the original ACEs study and collated from several other validated screening measures.^17,18^ Responses were characterized as binary variables: yes or no. ACEs were then reported retrospectively by young adults in the HA using a 10-item questionnaire from the original ACEs study.^17^ Because these surveys differed, we used the 8 overlapping items that were present in both surveys to conduct the analysis for this study. The number of ACEs was categorized into 4 groups (0 ACEs, 1 ACE, 2-3 ACEs, and ≥4 ACEs).

### Main Outcome: Sleep Quality

Sleep quality was assessed with the Pittsburgh Sleep Quality Index (PSQI) in young adulthood at the same time that retrospective ACEs were collected in the HA study.^19^ A summary score was generated by summing the 7 components of the PSQI, which included subjective sleep quality, sleep latency, sleep duration, habitual sleep efficiency, sleep disturbances, use of sleep medications, and daytime dysfunction. A binary variable was created to categorize scores greater than 5 as poor sleep and scores of 5 or lower as good sleep.

### Statistical Analysis

Statistical analysis was performed from January 2023 to January 2024. Univariable analyses were conducted in the overall sample to examine the distribution of ACEs and sociodemographic factors. Sociodemographic information included self-reported age (in years), sex (male or female), receipt of public assistance (yes or no), highest education grade completed, and geographical location in childhood (South Bronx or Puerto Rico). Separate multivariable linear regression models were used to examine the association of sleep quality with retrospective ACEs and prospective ACEs as both continuous and categorical variables, adjusting for covariates. Unstandardized β coefficients and 95% CIs were calculated from these models. A cross-product term between ACEs and the study site variable was included in the model to assess for interaction by site. Type III tests were used to examine statistical significance of the interaction term.

Similarly, log-binomial multivariable regression models were used to examine the association of retrospective and prospective ACEs with sleep quality as a dichotomous variable, adjusting for all covariates. Relative risks and 95% CIs were estimated from these models. All analyses included study sample weights to account for the unequal probability of selection into the study based on each site’s sampling design and to be representative of the age and sex distributions of the 2000 US Census. Nonresponse weights were calculated using a logistic regression model that included participant and parent characteristics associated with responding to the HA study. The final analysis weights were then calculated as a product of the HA nonresponse weights and the original BYS sampling weights to reflect the BYS reference population in each site. Analyses were conducted in SAS, version 9.4 (SAS Institute Inc). All *P* values were from 2-sided tests, and results were deemed statistically significant at *P* < .05.

## Results

Participant characteristics in the overall sample and by study site are shown in Table 1. In the sample of 813 participants, 438 (53.9%) lived in Puerto Rico as children, 411 (50.6%) identified as female, and 226 (27.8%) reported receiving public assistance. The mean (SE) age of participants was 22.9 (0.07) years, and the most frequent highest education grade completed was 12th grade (276 [34.2%]). A total of 343 participants (42.2%) identified as having a PSQI score of higher than 5, indicating poor sleep. A total of 584 participants (71.8%) reported having at least 1 prospective ACE, and 544 participants (66.9%) reported having at least 1 retrospective ACE. Compared with participants from Puerto Rico, those from the Bronx had, on average, worse sleep quality (mean [SE] PSQI, 5.7 [0.2] in the Bronx and 4.8 [0.2] in Puerto Rico), higher mean (SE) levels of prospective ACEs (1.4 [0.1] in the Bronx and 1.2 [0.1] in Puerto Rico), and higher mean (SE) levels of retrospective ACEs (1.7 [0.1] in the Bronx and 1.0 [0.1] in Puerto Rico).

**Table 1.** Participant Characteristics by Study Site, Health Assessment Study^a^

Variable	All participants (N = 813)	South Bronx (n = 375)	Puerto Rico (n = 438)
Sex			
Female	411 (50.6)	182 (48.5)	229 (52.3)
Male	402 (49.4)	193 (51.5)	209 (47.7)
Age, y			
18-23	508 (62.5)	170 (45.3)	283 (64.6)
24-29	305 (37.5)	205 (54.7)	155 (35.4)
Highest education grade completed			
6	2 (0.3)	0	2 (0.5)
7	5 (0.6)	1 (0.3)	4 (0.9)
8	6 (0.7)	4 (1.1)	2 (0.5)
9	17 (2.1)	12 (3.2)	5 (1.2)
10	33 (4.1)	31 (8.3)	2 (0.5)
11	46 (5.7)	42 (11.3)	4 (0.9)
12	276 (34.2)	129 (34.6)	147 (33.8)
College freshman	116 (14.4)	51 (13.7)	65 (14.9)
College sophomore	118 (14.6)	55 (14.8)	63 (14.5)
College junior	69 (8.5)	16 (4.3)	53 (12.2)
College senior	90 (11.1)	21 (5.6)	69 (15.9)
≥1 y of Graduate school	30 (3.7)	11 (3.0)	19 (4.4)
Receiving public assistance	226 (27.8)	70 (18.7)	156 (35.6)
Prospective ACEs			
None	229 (28.2)	87 (23.2)	142 (32.4)
1	302 (37.2)	143 (38.1)	159 (36.3)
2-3	241 (29.6)	125 (33.3)	116 (26.5)
≥4	41 (5.0)	20 (5.3)	21 (4.8)
Retrospective ACEs			
None	269 (33.1)	90 (24.0)	179 (40.9)
1	287 (35.3)	127 (33.9)	160 (36.5)
2-3	167 (20.5)	98 (26.1)	69 (15.8)
≥4	90 (11.1)	60 (16.0)	30 (6.9)
Individual prospective ACEs			
Verbal abuse	106 (13.4)	57 (15.7)	49 (11.4)
Physical abuse	158 (20.0)	76 (21.0)	82 (19.2)
Sexual abuse	35 (4.5)	18 (5.1)	17 (4.0)
Neglect	131 (16.5)	59 (16.1)	72 (16.8)
Divorce or separation	369 (46.9)	213 (60.2)	156 (36.0)
Domestic violence	19 (2.4)	7 (1.9)	12 (2.8)
Substance use in home	125 (15.5)	52 (14.0)	73 (16.7)
Maternal mental health issues	212 (26.2)	84 (22.6)	128 (29.4)
Individual retrospective ACEs			
Verbal abuse	122 (15.0)	80 (21.3)	42 (9.6)
Physical abuse	96 (11.8)	63 (16.8)	33 (7.5)
Sexual abuse	62 (7.6)	38 (10.2)	24 (5.5)
Neglect	24 (3.0)	21 (5.6)	3 (0.7)
Divorce or separation	464 (57.2)	248 (66.3)	216 (49.4)
Domestic violence	73 (9.0)	51 (13.6)	22 (5.1)
Substance use in home	140 (17.3)	92 (24.6)	48 (11.0)
Maternal mental health issues	120 (14.8)	56 (15.0)	64 (14.7)
PSQI score			
Good sleep (≤5)	470 (57.8)	192 (51.2)	278 (63.5)
Poor sleep (>5)	343 (42.2)	183 (48.8)	160 (36.5)

Abbreviations: ACEs, adverse childhood experiences; PSQI, Pittsburgh Sleep Quality Index.

^a^ Quality of evidence = 3, retrospective cohort study. Data are given as number (percentage) of participants.

A correlation analysis of retrospective and prospective ACEs showed a low but statistically significant correlation between these 2 measures (*r* = 0.134; *P* < .001). When prospective ACEs were examined as a categorical variable and adjusted for covariates, no level of ACEs was found to be significantly associated with sleep quality (Table 2). When examined as a continuous variable and adjusted for covariates, prospective ACEs were not found to have a significant association with sleep quality (β [SE] = 0.05 [0.10]; 95% CI, –0.14 to 0.24; *P* = .59) (Table 3).

**Table 2.** Association Between Prospectively Reported ACEs as a Categorical Variable and Sleep Quality^a^

Characteristic	Unadjusted model		Adjusted model^b^	
	β (SE) [95% CI]	*P* value	β (SE) [95% CI]	*P* value
No ACEs	0 [Reference]	NA	0 [Reference]	NA
1 ACE	0.17 (0.28) [−0.37 to 0.71]	.55	0.07 (0.28) [−0.47 to 0.61]	.80
2-3 ACEs	0.15 (0.29) [−0.42 to 0.72]	.61	0.04 (0.29) [−0.53 to 0.61]	.89
≥4 ACEs	0.36 (0.52) [−0.66 to 1.37]	.49	0.37 (0.51) [−0.64 to 1.38]	.47
From Puerto Rico	NA	NA	−0.96 (0.23) [−1.42 to −0.50]	<.001^c^
Female	NA	NA	0.44 (0.22) [−0.003 to 0.87]	.05
Age, y	NA	NA	0.01 (0.05) [−0.09 to 0.11]	.84
Highest education grade completed (3rd grade to graduate school)	NA	NA	0.02 (0.06) [−0.10 to 0.14]	.73
Public assistance	NA	NA	0.05 (0.26) [−0.47 to 0.57]	.85

Abbreviations: ACEs, adverse childhood experiences; NA, not applicable.

^a^ Quality of evidence = 3, retrospective cohort study.

^b^ Model controlled for site, sex, age, educational level, and receiving public assistance.

^c^ Significant at *P* < .05.

**Table 3.** Association Between Prospectively Reported ACEs as a Continuous Variable and Sleep Quality^a^

Characteristic	Unadjusted model		Adjusted model^b^	
	β (SE) [95% CI]	*P* value	β (SE) [95% CI]	*P* value
Total ACEs	0.07 (0.10) [−0.12 to 0.25]	.49	0.05 (0.10) [−0.14 to 0.24]	.59
From Puerto Rico	NA	NA	−0.96 (0.23) [−1.41 to −0.50]	<.001^c^
Female	NA	NA	0.43 (0.22) [−0.01 to 0.87]	.05
Age, y	NA	NA	0.01 (0.05) [−0.09 to 0.11]	.84
Highest education grade completed (3rd grade to graduate school)	NA	NA	0.02 (0.06) [−0.09 to 0.14]	.71
Public assistance	NA	NA	0.06 (0.26) [−0.46 to 0.57]	.83

Abbreviations: ACEs, adverse childhood experiences; NA, not applicable.

^a^ Quality of evidence = 3, retrospective cohort study.

^b^ Model controlled for site, sex, age, educational level, and receiving public assistance.

^c^ Significant at *P* < .05.

When retrospective ACEs were examined as a categorical variable and adjusted for covariates, higher levels of ACEs (≥2 ACEs) were significantly associated with worse sleep outcomes (2-3 ACEs: β [SE] = 1.25 [0.31]; 95% CI, 0.64-1.86; *P* < .001; ≥4 ACEs: β [SE] = 1.21 [0.38]; 95% CI, 0.47-2.0; *P* = .002) (Table 4). When retrospective ACEs were examined as a continuous variable and adjusted for covariates, they were found to be significantly associated with worse sleep outcomes (β [SE] = 0.29 [0.07]; 95% CI, 0.15-0.44; *P* < .001) (Table 5). In addition, these results did not vary significantly by site.

**Table 4.** Association Between Retrospectively Reported ACEs as a Categorical Variable and Sleep Quality^a^

Characteristic	Unadjusted model		Adjusted model^b^	
	β (SE) [95% CI]	*P* value	β (SE) [95% CI]	*P* value
No ACEs	0 [Reference]	NA	0 [Reference]	NA
1 ACE	0.43 (0.26) [−0.08 to 0.95]	.10	0.38 (0.26) [−0.14 to 0.89]	.16
2-3 ACEs	1.46 (0.31) [0.86 to 2.06]	<.001^c^	1.25 (0.31) [0.64 to 1.86]	<.001^c^
≥4 ACEs	1.46 (0.37) [0.72 to 2.19]	<.001^c^	1.21 (0.38) [0.47 to 1.96]	.002^c^
From Puerto Rico	NA	NA	−0.74 (0.24) [−1.20 to −0.27]	.002^c^
Female	NA	NA	0.30 (0.22) [−0.13 to 0.74]	.17
Age, y	NA	NA	0.008 (0.05) [−0.09 to 0.11]	.88
Highest education grade completed (3rd grade to graduate school)	NA	NA	−0.004 (0.06) [−0.12 to 0.11]	.95
Public assistance	NA	NA	0.04 (0.26) [−0.48 to 0.55]	.89

Abbreviations: ACEs, adverse childhood experiences; NA, not applicable.

^a^ Quality of evidence = 3, retrospective cohort study.

^b^ Model controlled for site, sex, age, educational level, and receiving public assistance.

^c^ Significant at *P* < .05.

**Table 5.** Association Between Retrospectively Reported ACEs as a Continuous Variable and Sleep Quality^a^

Characteristic	Unadjusted model		Adjusted model^b^	
	β (SE) [95% CI]	*P* value	β (SE) [95% CI]	*P* value
Total ACEs	0.36 (0.07) [0.21 to 0.50]	<.001^c^	0.29 (0.07) [0.15 to 0.44]	<.001^c^
From Puerto Rico	NA	NA	−0.76 (0.24) [−1.22 to −0.30]	.001^c^
Female	NA	NA	0.32 (0.22) [−0.12 to 0.76]	.15
Age, y	NA	NA	−0.005 (0.05) [−0.11 to 0.10]	.92
Highest education grade completed (3rd grade to graduate school)	NA	NA	0.003 (0.06) [−0.11 to 0.12]	.96
Public assistance	NA	NA	0.03 (0.26) [−0.48 to 0.54]	.91

Abbreviations: ACEs, adverse childhood experiences; NA, not applicable.

^a^ Quality of evidence = 3, retrospective cohort study.

^b^ Model controlled for site, sex, age, educational level, and receiving public assistance.

^c^ Significant at *P* < .05.

When the PSQI was examined as a binary variable, participants with a greater number of retrospective ACEs were more likely to have poor sleep quality compared with those with fewer retrospective ACEs, after adjusting for sociodemographic factors (adjusted risk ratio, 1.22; 95% CI, 1.06-1.42). Prospective ACEs were not found to have a statistically significant association with poor sleep quality as a binary variable.

## Discussion

In this cohort of 813 Puerto Rican young adults, retrospective ACEs were significantly associated with poor sleep quality, and prospective ACEs were not significantly associated with poor sleep quality, after controlling for sociodemographic factors. There are several hypotheses that may explain these findings. Research suggests that ACEs are associated with a stress and fear response that can lead to disruption in regular routines and sleep and circadian dysregulation.^17^ The difference in associations seen with prospective and retrospective ACEs may be due to the fact that prospective ACEs were gathered over the first 11 years of the child’s life, resulting in a gap in reporting during the adolescent period. This gap could explain the appearance of overreporting in young adulthood, which may represent events that occurred during the adolescent period.^20^ Furthermore, research shows that retrospective ACEs are strongly associated with subjectively measured outcomes, such as the PSQI.^21^ Participants with retrospective ACEs could have demonstrated more enduring effects of ACEs, resulting in worse sleep outcomes in young adulthood compared with those who did not retrospectively report ACEs.

### Strengths and Limitations

There are several strengths to this study. Most studies on ACEs and sleep quality focus on Asian and White populations, but our study diversified the Hispanic or Latino diaspora by focusing on Puerto Ricans. The geographical diversity of this cohort allowed us to examine whether social context may modify the association between ACEs and sleep quality. Furthermore, our study prioritized an understudied age group to highlight the specific circumstances of young adults, which has potential clinical relevance for supporting the pediatric-to-adult transition in medical care. Other strengths of this study include the large sample size, longitudinal design, and high compliance rate at follow-up. This study also contributes to the growing body of evidence that supports the utility of both retrospective and prospective ACEs when assessing overall health.^11^

Despite these strengths, there are some limitations of our study. Although the PSQI is a validated survey, it is a self-reported measure that has been shown to poorly correlate with objective measures of sleep quality, such as polysomnography.^22,23,24^ Retrospective ACEs do not capture the exact timing of an event, while prospective ACEs provide a more specific timeline. Recall bias is a consideration when observing the results from the retrospective measures, although research shows no evidence of recall bias in the retrospective assessment.^25^ There is also the possibility of reporter bias with retrospective ACEs, as the same informant is reporting on the exposure and outcome at the same time. However, reporter bias from retrospective ACEs has been shown to have the potential to both overestimate and underestimate subjective health outcomes, so the association of this bias in our study is indeterminant.^21^ It is also possible that prospective ACEs are underreported in cases of harm to a child.^21^ In addition, the ACE questionnaires used in this study give equal weight to each ACE, which does not take into account descriptive measures, such as frequency and chronicity of ACEs. Finally, because our study focused on a population of young adults in 2 urban settings, future studies should exercise caution when generalizing these findings to other demographics.

## Conclusions

In this cohort study, Puerto Rican young adults who retrospectively reported higher numbers of ACEs were more likely to have worse sleep quality when controlling for sociodemographic factors, while no significant association was found between prospective ACEs and sleep quality. This study suggests that prospective and retrospective ACEs are important metrics of overall health and may be a useful screening tool for understanding sleep health among young adults. Subsequent studies should include objective measures of sleep quality and weighted ACE questionnaires and should examine specific pathways that may explain the association between ACEs and sleep disturbances.

## Notes

Supplement.
Data Sharing Statement
