# Supplementary material for: Adverse Childhood Experiences and Sleep Disturbances Among Puerto Rican Young Adults
Source: JAMA Netw Open. 2024 Apr 22;7(4):e247532. doi: 10.1001/jamanetworkopen.2024.7532 (PMC11036138; doi:10.1001/jamanetworkopen.2024.7532)
Supplement: Supplement. — Data Sharing Statement [file jamanetwopen-e247532-s001.pdf]

Data Sharing Statement

Olsen. Adverse Childhood Experiences and Sleep Disturbances Among Puerto Rican Young Adults. *JAMA Netw Open*. Published April 22, 2024. doi:10.1001/jamanetworkopen.2024.7532

Data
Data available: No. The authors decline to share the data used in this study. Please direct all inquiries to the study investigators.
